# Longitudinal extensive transverse myelitis after chemoradiation therapy with durvalumab, a rare complication: case report

**DOI:** 10.1186/s12883-022-02576-7

**Published:** 2022-03-19

**Authors:** Travis Moodie, Ola Alshaqi, Abdul Alchaki

**Affiliations:** 1 University of South Dakota Sanford School of Medicine, Sioux Falls, SD USA; 2 Damascus University, Damascus, Syria

**Keywords:** Durvalumab, Longitudinal extensive transverse myelitis

## Abstract

**Background:**

Longitudinal extensive transverse myelitis is a rare and potentially life-threatening complication of chemoradiation. Certain chemotherapy agents have been proposed to increased neurotoxicity with chemoradiation therapy. One such agent is durvalumab, a human IgG1 monoclonal antibody that blocks programmed death ligand 1, allowing T-cells to recognize and kill tumor cells. Durvalumab and other immune checkpoint inhibitors may also cause transverse myelitis without concomitant treatment with radiation. Durvalumab is a standard therapy for non-small cell lung carcinoma. Here we present a case of a 68-year-old male who presented after chemoradiation and durvalumab therapy with transverse myelitis extending outside the irradiation site.

**Case presentation:**

A 68-year-old male presented to the emergency department with pain and weakness in his feet and hesitancy of urination. Medical history is significant for non-small cell lung cancer treated with chemoradiotherapy and consolidation therapy with durvalumab for one year. His last radiation treatment was 15 months prior, and his last infusion of durvalumab was 3 months prior. Exam revealed severe weakness of bilateral legs with absent vibration sensation. MRI showed central longitudinal extensive transverse myelitis extending from C4-T11. CSF studies showed 8 WBC with 63% lymphocyte predominance and a protein of 48. Oligoclonal bands and angiotensin-converting enzyme were negative. Serum Neuromyelitis Optica antibody (AQP4-IgG) and Myelin oligodendrocyte glycoprotein antibody (MOG-IgG) were negative. Infectious workup came back negative. The patient was treated with steroids and plasma exchange with mild improvement. Etiology remained unknown, but longitudinal extensive transverse myelitis following durvalumab chemoradiotherapy was thought to be the likely cause. He was discharged on a high-dose prednisone taper with outpatient follow-up. His condition worsened near the end of the steroid taper. High-dose prednisone and cyclophosphamide infusions were started with mild improvement and stabilization of the patient’s condition. He transitioned to methotrexate after completion of six cyclophosphamide infusions. The patient expired due to complications from his cancer.

**Conclusion:**

Longitudinal extensive transverse myelitis is a rare and potentially life-threatening complication of durvalumab therapy. As durvalumab has become a standard treatment for non-small cell lung cancer, it is important to be able to identify and treat side effects.

## Background

Myelopathy is described as any pathological process affecting the spinal cord [1]. These processes can be divided into two groups: compressive and noncompressive. Noncompressive causes can be further divided into vascular, inflammatory, and other unique etiologies [2]. Longitudinal extensive transverse myelitis (LTEM) defined by a spinal MRI indicating a lesion extending over three or more vertebral segments. While rare, Longitudinal extensive transverse myelitis can lead to catastrophic morbidity. LTEM is classically associated with neuromyelitis optica. Other causes include inflammatory etiologies, infection, malignancy, vascular and radiation therapy. Radiation myelitis is typically a subacute and progressive process that presents with symptoms nine to fifteen months after completion of radiation therapy. Concomitant use of certain chemotherapy agents has been associated with increased neurotoxicity [3, 4]. One such postulated chemotherapy agent is durvalumab. Durvalumab is a human IgG1 monoclonal antibody that blocks programmed death ligand 1 (PD-L1) binding to programmed death 1 (PD-1) and CD80, allowing T cells to recognize and kill tumor cells [1, 5]. Durvalumab and other immune checkpoint inhibitors have been associated with LTEM without radiation therapy [6]. Durvalumab became a standard consolidation treatment for non-small cell lung cancer after chemoradiation therapy when it demonstrated a survival benefit in the PACIFIC trial. Here we present a case of a 68-year-old male presenting with longitudinal extensive transverse myelitis after chemoradiation therapy with durvalumab.

## Case presentation

A 68-year-old male present to the emergency department with pain and weakness in his feet. Medical history is significant for non-small cell lung cancer treated with chemoradiotherapy (Taxol and carboplatin) and consolidation therapy with durvalumab for one year. Five days prior he was diagnosed with neuropathy by his oncologist for tingling and numbness in his feet. Three days prior he became progressively weaker to the point where he could not stand, and he lost bowel and bladder function. He described the pain as pins and tingling throughout his leg. Pain was worse with gentle touch. He had hesitancy and frequency of urination. He denies back pain, trauma, or fever. His last radiation treatment was 15 months prior. Radiation was delivered over the left lung and mediastinum. The patient received 2 Gy doses for 31 treatments over a 45-day period for a total dose of 62 Gy. His last durvalumab infusion was 3 months prior. Exam revealed a low-grade temp of 99.3 °F with other vitals stable. General medical exam was unremarkable. Neurological exam showed muscle contraction without joint movement throughout the lower extremities, bilateral diminished patellar and Achilles reflexes, and a non-tender back. He was admitted to the hospital for additional management.

**Fig. 1:**
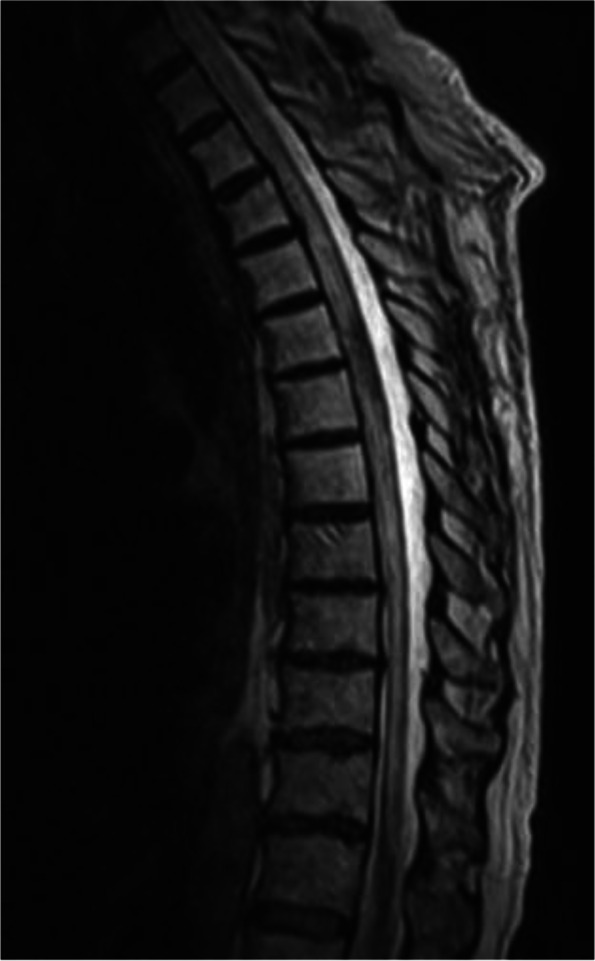
MRI thoracic spine; Sagittal T2. Showed extensive T2 hyper intense signal (C5 till T11)

**Fig. 2:**
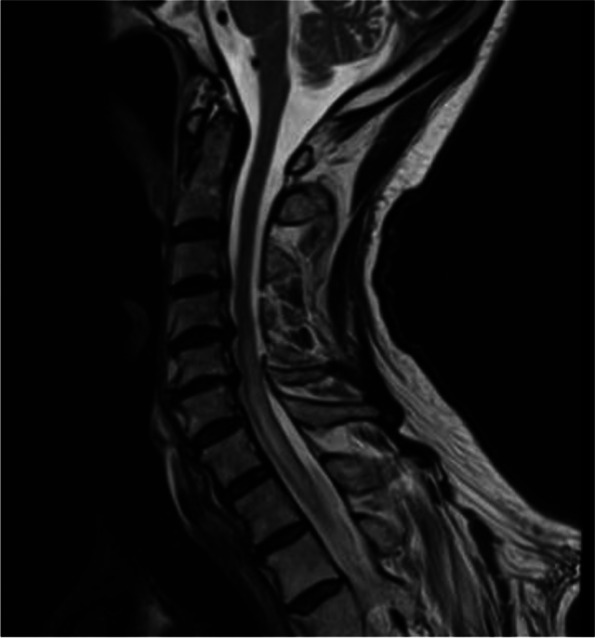
MRI cervical spine: sagittal T2: hyper intense intra-medullary T2 signal

**Fig. 3:**
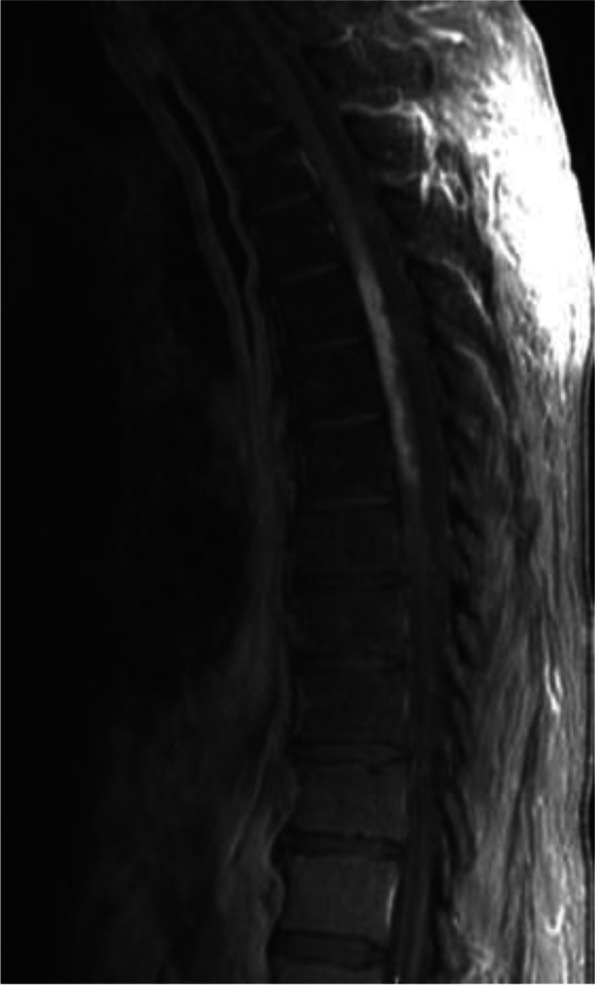
MRI thoracic spine, Sagittal T1 with Gad: showed an enhancing lesion from T3 till T7

Brain MRI displayed chronic small vessel disease. Spinal MRI showed abnormally increased spinal cord signal intensity without cord expansion on T2-weighted images from C4-T11 suggestive of longitudinal extensive transverse myelitis (LETM) (Figs. 1 and 2). T1 post Gadolinium images showed enhancement at T5-T6 (Fig. 3). CSF studies revealed 8 WBC with 63% lymphocyte predominance and a protein of 48. Oligoclonal bands were negative with 3 bands in the serum and 3 bands in the CSF. Angiotensin-converting enzyme was negative as well. Viral etiologies including cytomegalovirus, enterovirus, herpes simplex virus 1 and 2, varicella-zoster virus, and West Nile were all negative. Serum Neuromyelitis Optica antibody (AQP4-IgG) and Myelin oligodendrocyte glycoprotein antibody (MOG-IgG) were negative. An autoimmune panel including Sjogren, anti-Smith antibody, scleroderma, and double DNA antibodies were negative. He had a positive antinuclear antibody with titration of 1:1280. Mayo clinic’s paraneoplastic autoantibody panel also came back negative.

The etiology remained unknown. The differential diagnosis included seronegative neuromyelitis optica, paraneoplastic syndrome, or autoimmune reaction secondary to Durvalumab with radiation. The patient was placed on high-dose steroids and plasma exchange with mild improvement in symptoms. He was discharged on a steroid taper and planned for outpatient follow-up at a neurology clinic in 3 months.

He finished his steroid taper the day before his clinic visit. At the clinic, he reported worsening bilateral leg weakness over the last few weeks. No muscle contraction was observed in the left side, and motion on the right was limited by gravity. He had decreased vibration sensation in both legs and decreased temperature sensation in his right leg. He was placed on 40 mg of prednisone daily and a repeat MRI was scheduled for 1 week later.

**Fig. 4:**
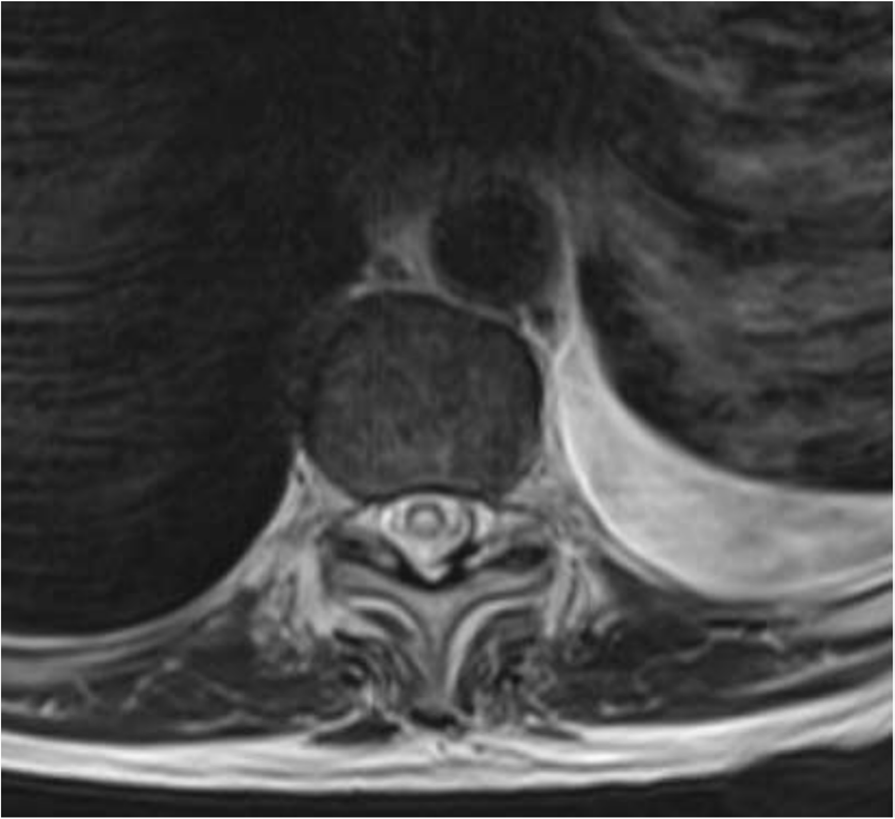
MRI thoracic spine, axial T2: showed central intra medullary T2 hyper intense signal

**Fig. 5:**
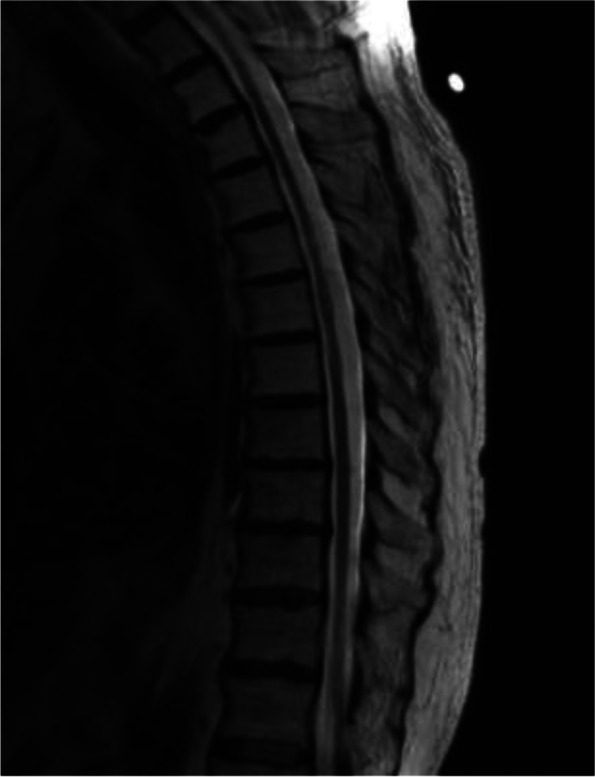
MRI thoracic spine: Sagittal T2: After 6 cycles of cyclophosphamide treatment: resolving of T2 signal. Atrophy of the spinal cord

One day before his MRI, he called the clinic due to new leg stiffness further limiting mobility and no clinical improvement from the prednisone. His prednisone was increased to 80 mg with plans to further evaluate after his MRI. MRI showed worsening T2 hyperintense signal, particularly over the thoracic region (Fig. 4). The decision was made to start cyclophosphamide Euro-lupus protocol, a 500 mg dose of cyclophosphamide IV given every two weeks for a total of six doses [7], in addition to increasing his prednisone to 100 mg daily. He saw mild improvement in leg weakness after the initial infusion. Infusions were continued every two weeks for a total of 6 doses, and the steroid was slowly tapered. The patient had no new or worsening neurological conditions during this time. The patient was started on oral methotrexate 7.5 g weekly with daily folic acid after his fifth cyclophosphamide infusion. Repeat MRI showed near complete interval resolution of the significant intramedullary increased T2 signal in the cervical spinal cord from C4 to T4 and no enhancement, but there was residual atrophy of the spinal cord (Fig. 5). Methotrexate was increased to 10 mg weekly. Shortly after, the patient was hospitalized with radiation pneumonitis. He transitioned to comfort care and expired within the month.

## Discussion and conclusions

Longitudinal extensive transverse myelitis is a rare and potentially life-threatening condition. Symptoms include pain, sensory deficits, motor deficits, and bladder and rectal disturbances. The MRI pattern seen in our patient is classically associated with Neuromyelitis Optica Spectrum Disorder (NMOSD), Myelin oligodendrocyte glycoprotein antibody disorders, Acute disseminated encephalomyelitis, infectious myelitis, Dural arteriovenous fistula, and radiation [1]. Therefore, a thorough workup with CSF studies, infectious panels, AQP4-IgG, MOG-IgG, and additional imaging as needed should be completed to help exclude these diagnoses as well as a paraneoplastic antibody panel to rule out paraneoplastic syndromes. Prior cases of patients treated with immune checkpoint inhibitors that develop LETM have demonstrated reactivity with Anti-AQP4, Anti-GFAP, Anti-CV2, Anti-CRMP5 antibodies, but these are usually negative [8, 9]. Nonetheless, a positive Anti-AQP4 may make it difficult to discern between NMOSD and LTEM due to immune checkpoint inhibitors.

Another case report by Kubo K et. all presents a patient with radiation myelitis after durvalumab therapy [3]. Their patient also had difficulty urinating and lower extremity strength and sensory deficits. However, their patient developed symptoms more rapidly after chemoradiotherapy than ours; 2.5 months versus 15 months. Their patient’s MRI results also showed the myelitis was contained within the irradiated site while our patient’s MRI results showed diffused myelitis extending outside the irradiated site. Their patient received a total dose of 40 Gy delivered as 2 Gy doses for 15 treatments followed by 1 Gy doses for 10 treatments, much less than the 62 Gy our patient receive. This is a significant difference as Schultheiss T. demonstrated a cumulative dose of 45 Gy was associated with a 0.02% myelopathy while a dose of 59.3 Gy delivered a 5% chance of myelopathy when delivered [10]. It should be noted these numbers were determined for radiation-induced cervical myelopathy, and the same study note decreased radiation sensitivity to the thoracic spinal cord. Lastly, both patients had a positive response to steroid therapy but also required plasma exchange on initial presentation. Our patient was also placed on long-term immunosuppressants after worsening of symptoms while weaning off steroids. Unfortunately, we do not know if the Kubo K. et. all. Patient had a similar long-term course as the paper only reported out a one-week follow-up.

The pathophysiology remains unclear. Potential mechanisms as outlined by Oliveira et. all include the following: a shift toward the pro-inflammatory profile of T lymphocytes dominated by Th1/Th17 differentiation that increases the production of pro-inflammatory cytokines, autoreactive antibody production, activation of potentially pre-existing self-reactive T cells, and a cross-reactivity between normal tissue antigens and tumor neo-antigens [8]. However, further research is needed in this area to identify the exact mechanism.

This case report demonstrates a rare and potentially devastating complication of chemoradiation therapy followed by durvalumab. As durvalumab has become more standard in the treatment of non-small cell lung cancer, it is important to recognize and treat potential side effects of the medication.

## Data Availability

Not applicable.

## Abbreviations

CSF: Cerebrospinal fluid
MRI: Magnetic resonance imaging
